# Falls associated with indoor and outdoor environmental hazards among community-dwelling older adults between men and women

**DOI:** 10.1186/s12877-021-02499-x

**Published:** 2021-10-12

**Authors:** Sungmin Lee

**Affiliations:** Department of Landscape Architecture and Urban Planning, Texas A&M University, 3137 TAMU, College Station, TX 77840 USA

**Keywords:** Gender differences, Indoor and outdoor environmental hazards, Clinical and environmental fall prevention

## Abstract

**Background:**

Hazardous environmental exposures are recognized risk factors for falls among older adults. However, the gender differences in the associations of falls with indoor and outdoor environmental hazards are scarce. This study examined the indoor and outdoor environmental risk factors for falls and compared the data for men and women among U.S. older adults using nationally representative data.

**Methods:**

We used the 2011 National Health and Aging Trends Study (NHATS) for a cross-sectional analysis of 6680 community-dwelling adults aged ≥65 years in the United States. A series of logistic regressions was used to identify the indoor and outdoor environmental hazards associated with falls stratified by gender after adjusting for sociodemographic, health, and behaviors. We also tested for significant interactions with gender.

**Results:**

Compared to men, women had a higher prevalence of falls. In the model adjusted for sociodemographic, health, and behavioral conditions, there were gender differences in the association of falls with the presence of indoor and outdoor environmental hazards. Gender-specific analyses showed that women with the presence of indoor environmental hazards (OR = 1.37, 95% CI = 1.04.-1.79) had higher odds of falls, whereas for men, the presence of outdoor environmental hazards (OR = 1.34, 95% CI = 1.02–1.75) was associated with falls. We also found a significant interaction term between outdoor environmental hazards and gender (OR = 0.65, 95% CI = 0.47–0.90). The interaction plot indicated that the presence of outdoor environmental hazards increased the risks of falling in men but not in women.

**Conclusions:**

Significant gender differences exist in the association of falls with indoor and outdoor environmental hazards among older men and women. Our findings suggest that gender-tailored prevention programs to increase awareness of the environmental hazards and gender-specific environmental interventions are needed to help prevent falls.

**Supplementary Information:**

The online version contains supplementary material available at 10.1186/s12877-021-02499-x.

## Introduction

Falls among older adults have received considerable attention as a major public health concern in the United States. Approximately one-fourth of community-dwelling adults aged 65 years or older in the United States experience falls each year [1]. Among those who fall, one-third suffer serious injury, including hip fractures and traumatic brain injury that may require hospitalization [2]. Injuries from falls were shown to result in various individual health and behavior outcomes, including functional decline, loss of independence, fear of falling, and social isolation [3].

Falls among older adults are a result of complex factors, involving individual and environmental circumstances [4, 5]. Individual factors for risk of falling include sociodemographic, health, and behavioral characteristics. For example, older adults who have functional limitations, muscle weakness, comorbidities, anxiety, and lack of physical activity tend to be exposed to the risk of falling [6]. Environmental factors refer to all attributes that are external to the human host, which include slippery or uneven surface, obstacles, stairs, abrupt vertical transitions, and weather condition [7]. These individual and environmental factors are known to be independently associated with the occurrence of falls [8].

In terms of individual risk factors for falls, gender is frequently reported as a risk factor of falling [9]. The majority of studies reported higher rates of falling in women than men [10, 11]. These gender disparities in fall are known to be associated with differences in a wide range of health, behavioral, and psychosocial factors. For example, women reported poor self-rated health conditions, vision impairment, and deterioration in muscle and bone mass, which makes females more vulnerable to risks of falling [12]. Other gender differences in risk factors for falling include sleep deprivation [13], diabetes [14], and vitamin D deficiency [15]. In addition, perceptions about falling may be different by gender. Men tend to underreport their challenges related to falling and report higher self-efficacy related to falls, compared to women. Men were also more likely to be reluctant to admit having a fear of falling, compared to women [16].

Several studies have identified the importance of the home settings and neighborhood environments for individual health and well-being of older adults [17]. Most older adults prefer to age in place in their homes and neighborhoods. As people age with reduced mobility and functional loss, they tend to spend more time in the home and in their communities. Especially for community-dwelling older adults, they could be directly and indirectly influenced by home and neighborhood environments that include barriers and problems or lack supportive features. Common barriers in the home environment include poor flooring conditions, poorly designed tubs, badly arranged furniture, inadequate lighting, and obstructed walkways, all of which potentially increase fall risks [18].

Besides the environmental barriers in the home, several outdoor environment features in the neighborhood including bumpy walking surfaces, steep curb ramps, and poorly maintained street conditions have been identified as barriers that limit activity [19]. For example, older adults who reported poorly maintained street conditions in their neighborhoods have more activity limitations, while those living in areas with better public transportation and accessible parks have less activity limitations [20]. Relatively limited studies, have identified the neighborhood environment as a risk factor of falls. Uneven surfaces on streets have also been reported as a risk factor of falls [21, 22]. Qualitative studies showed that older individuals reported uneven walking surfaces, inadequate maintenance, poor lighting, and traffic patterns as perceived risk factors [23].

Another line of research has classified fall risk factors into those that can cause indoor and outdoor falls. Previous studies have shown that older adults at high risk for outdoor falls were different from those at high risk for indoor falls [24]. Indoor falls tended to occur among frail individuals while outdoor falls were more likely to occur among more active people with healthier characteristics. Specifically, Kelsey et al. [25] found that older participants with poor baseline health characteristics had elevated rates of indoor falls while healthy and younger older adults had elevated rates of outdoor falls. Previous studies also showed different consequences of indoor and outdoor falls [26]. Mänty, Heinonen [27] found that indoor falls were associated with future mobility limitations in Finnish women while outdoor falls were not.

The circumstances surrounding indoor and outdoor falls may also differ by age and gender. Compared to outdoor fallers, indoor fallers were more likely to be women and older [28]. In the location-specific analyses, women reported higher fall rates in the kitchen while performing household activities, but there were no gender differences in their rates of outdoor falls (Duckham et al., 2013). Although the exact mechanism of why there were gender differences in the likelihood of falls is still unclear, men and women differ in their time spent indoors and outdoors, which could potentially result in different fall tendencies in different locations [29]. For example, older women are more likely to spend time doing housework inside the home compared to older men [30]. Although men have tended to spend more time inside the home and have tended to do more housework after retirement in recent years [31], the types of household labor are still unequal between older men and women. Specifically, older women are typically responsible for routine and repetitive tasks such washing dishes, preparing meals, and cleaning the house inside the house while men engage in more occasional tasks, such as household repairs, yard work, and vehicle maintenance, which is done more outside the house [32]. These differences should be considered when developing prevention programs and environmental interventions for falls.

Although numerous studies have identified risk factors of falling and even environmental risk factors for falls, few studies have comprehensively examined how indoor and outdoor environmental hazards are associated with increased falls simultaneously. Additionally, no study has been conducted to understand such relationship across genders. To address these concerns, we analyzed the prevalence of falls in relation to the environmental hazards inside home and outside home environments among community-dwelling individuals aged 65 or older by gender. Since women and men have different activities and social roles within the home and neighboring areas, we hypothesized that the men’s and women’s prevalence of falls were differently associated with environmental factors in locations. The present study contributes to the literature by improving our understanding of environmental risk factors for falls by gender. The findings will be useful for creating tailored and optimal fall prevention strategies for both male and female older adults.

## Methods

### Study design, setting, and sample

The present study investigated data from the National Health and Aging Trends Study (NHATS), which is an ongoing longitudinal study that surveys a nationally representative sample of 35.3 million Medicare beneficiaries aged 65 or older who reside in the United States [33]. The sample frame of NHATS relies on Medicare enrollment and receives funding from the National Institute on Aging (NIA). Data are collected by trained personnel through annual in-person interviews and assessments. Among 12,411 selected individuals, 8245 older adults participated in the survey in 2011 (71% response rate). This study excluded respondents who resided in nursing homes (*n* = 468) or similar settings (*n* = 412) and non-self respondents (*n* = 517). The final study sample of this study was 6680 community-dwelling older adults.

### Measures

The presence of having one or more than one fall in 12 months was assessed as an outcome. In the survey, the participant was asked to report if he or she had fallen down in the past 12 months. In this study, a fall was defined as reporting one fall, slip or trip in which they lost their balance and land on the floor or ground or at a lower level during the past year. This definition was consistent across the studies [34].

Environmental hazard variables were assessed through interviewer observations using an environmental checklist. In this study, environmental hazard variables were assessed in two dimensions: (a) indoor environments *(*e.g.*, kitchen, living room within home settings)*; (b) *outdoor environments (*e.g.*, garden, access path, streets, or sidewalks outside home)*. Each indoor environmental hazard variable was specifically measured using a dichotomous response (“no” and “yes”) to three items: 1) broken furniture or lamps, 2) flooring in need of repair, and 3) other tripping hazards (e.g., pathways not clear, throw rugs not secured, electrical cords in path). Other dichotomous responses were included to measure outdoor environmental hazards based on the following questions: “Standing in front of the individual home/building, did it have 1) a crumbling foundation or open holes, and 2) uneven walking surfaces or broken steps in the area leading to the home/building?” The final variable of outdoor environments was 3) litter, broken glass, or trash on sidewalks and streets, measured by interviewers when they looked around in every direction of the participant’s home/building. This item was initially measured using a four-scale response, which was collapsed into a dichotomous variable (“none” and “a little, some, and a lot”) to facilitate the comparison with other environmental variables.

Given the multiple variables for environmental hazards causing the potential multicollinearity issue, we attempted to construct a single variable by combining all variables within each domain (indoor and outdoor). In the study, we constructed a dichotomous variable for each indoor and outdoor environmental hazards (no hazard at all vs. one or more hazards) due to the skewed distribution and straightforward interpretation. As a robust check, we also tested a series of categorical variable (no hazard at all, one, two, and three hazards) and index by using a principal component analysis (PCA). A PCA was performed in Stata using varimax rotation based on an eigenvalue of 1. All variables were considered to load as the factor loading of each variable was at least .40. Including these different independent variables in the models provided similar results with similar model fit, which was reported in Additional file 1: Appendix Table 1.

We included a series of covariates, which were selected based on previous literature about risk factors for falling [6]. The covariates included sociodemographic, health, and behavioral factors. The sociodemographic variables included the following seven variables: age group (65–74, 75–84, 85+ years old), gender (female and male), race/ethnicity (non-Hispanic White and others), education (less than high school diploma, high school graduate, some college, and bachelor’s degree or advanced degree), job status (no job or retirement versus job), home ownership, household income (< $25,000 vs. ≥ $25,000) and house type (single home, duplex or townhome, mobile home, and multi-family home). The health factors included self-reported poor health conditions, fear of falling in the last month (yes or no), balance impairment in the last month (yes or no), mobility limitations within a quarter-mile (yes or no), vision impairment (yes or no), and arthritis (yes or no). The number of medical conditions was also measured based on the following medical conditions: osteoporosis, heart disease, cancer, diabetes, lung disease, arthritis, and stroke, which collapsed into a single variable (no problem, one, two, three, or four and more). Additionally, we included self-reported depression (yes or no), which was measured using the responses (none, several days of feeling depressed, more than half of the days of feeling depressed, or nearly every day of feeling depressed). We also included frequency of taking pain medication in the past month (every and most days vs. some days, rarely, and never). For walking-related behaviors, we used gait speed, which was calculated using distance in meters and time in seconds and used part of the short physical performance battery test [35]. Participants were instructed to walk over a 3-m course from a standing start at their usual pace, with the faster of two-timed trials used. We also used three dichotomous questions about the use of a walking aid when walking outside the home (yes or no) and the frequency of outside travel in the last month (5 or more days per week versus 0–4 days per week), and experience of walking for exercise (yes or no).

### Statistical analysis

Descriptive results are presented for each gender subgroup to understand the differences in the sociodemographic, health, behavioral, and environmental characteristics (Table 1). Statistical tests were applied to investigate whether gender differences are statistically significant. A chi-square test and t-test were used for each categorical variables and continuous variables. Bivariate logistic regression models stratified by gender were then used to estimate the odds ratios (ORs) and 95% confidence intervals (CI) of falls for each risk factor (Table 2). All risk factors associated with falls from the bivariate test were included in the multivariable models and were tested (Table 3). We also checked for multicollinearity based on the variance inflation factor (VIF) and found no multicollinearity issue (all VIFs were less than 4). Finally, we considered the interaction terms for gender with the number of built environmental hazards in different locations (Table 4). These analyses were performed to identify the potential interactions of gender with indoor and outdoor environmental hazards. All statistical analyses were performed using Stata IC 15.0 (Stata Corp, College Station, TX). For the model fit for testing interaction term, we used Akaike’s information criterion (AIC), with a smaller value indicating a better fitting model.

**Table 1:** Characteristics of Study Participants

	Total (***N*** = 6680) Frequency (%)	Men (***N*** = 2845) Frequency (%)	Women (***N*** = 3835) Frequency (%)	***P***-value
Falls	1975 (29.60%)	756 (26.61%)	1219 (31.82%)	< 0.001
*Environmental hazards*				
Indoor environmental hazards (1+ hazards)	860 (13.73%)	363 (13.86%)	496 (13.64%)	0.803
Outdoor environmental hazards (1+ hazards)	1378 (21.88%)	574 (21.73%)	804 (21.98%)	0.816
*Sociodemographic*				
Age				< 0.001
65–74	2846 (42.60%)	1286 (45.20%)	1560 (40.68%)	
75–84	2692 (40.30%)	1127 (39.61%)	1565 (40.81%)	
85+	1142 (17.10%)	432 (15.18%)	710 (18.51%)	
Non-Hispanic White	4607 (68.97%)	1988 (69.88%)	2619 (68.29%)	0.166
Education				< 0.001
Less than high school diploma	1694 (25.62%)	717 (25.44%)	977 (25.74%)	
High school graduate	2311 (34.95%)	827 (29.35%)	1484 (39.10%)	
Some college	1164 (17.60%)	462 (16.39%)	702 (18.50%)	
Bachelor’s degree or advanced degree	1444 (21.84%)	812 (28.81%)	632 (16.65%)	
No job or retirement (yes vs. no)	5789 (87.89%)	2361 (84.14%)	3428 (90.66%)	< 0.001
Household income (≥ $25,000 vs. <$25,000)	3652 (54.67%)	1877 (65.98%)	1775 (46.28%)	< 0.001
Home ownership (Own vs. rent)	5013 (76.24%)	2250 (80.21%)	2763 (73.29%)	< 0.001
House type				< 0.001
Single house	5043 (75.73%)	2221 (78.34%)	2822 (73.80%)	
Duplex and townhouse	538 (8.08%)	201 (7.09%)	337 (8.81%)	
Mobile home	327 (4.91%)	146 (5.15%)	181 (4.73%)	
Multi-family house	751 (11.28%)	267 (9.42%)	484 (12.66%)	
*Health*				
Poor health condition (yes vs. no)	456 (6.83%)	198 (6.96%)	258 (6.73%)	0.716
Fear of falling (yes vs. no)	1862 (27.88%)	607 (21.34%)	1255 (32.72%)	< 0.001
Balance impairment (yes vs. no)	1925 (28.83%)	685 (24.08%)	1240 (32.35%)	< 0.001
Mobility limitation within a quarter mile (yes vs. no)	1905 (28.68%)	552 (19.47%)	1353 (35.53%)	< 0.001
Vision impairment (yes vs. no)	557 (8.34%)	193 (6.78%)	364 (9.49%)	< 0.001
Arthritis (yes vs. no)	3680 (55.18%)	1291 (45.44%)	2389 (62.41%)	< 0.001
Number of medical conditions				< 0.001
0	594 (8.98%)	316 (11.20%)	278 (7.32%)	
1	1209 (18.27%)	584 (20.70%)	625 (16.46%)	
2	1707 (25.80%)	722 (25.59%)	985 (25.95%)	
3	1523 (23.02%)	601 (21.30%)	922 (24.29%)	
4+	1584 (23.94%)	598 (21.20%)	986 (25.97%)	
Depression (Several days, more than half the days, nearly every day vs. non)	1867 (27.99%)	633 (22.29%)	1234 (32.21%)	< 0.001
Medication use (every and most days vs. some days, rarely, and never)	1715 (25.70%)	624 (21.95%)	1091 (28.49%)	< 0.001
*Walking-related behavior*				
Gait speed (m/s)^a^	0.93 (0.39)	1.00 (0.39)	0.88 (0.38)	< 0.001
Use of a walking aid (yes vs. no)	1867 (27.99%)	633 (22.29%)	1234 (32.21%)	< 0.001
Frequency of going outside (5 days and more a week vs. less than 5 days a week)	5646 (84.55%)	2603 (91.53%)	3043 (79.37%)	< 0.001
Walk for exercise (yes vs. no)	3956 (59.23%)	1804 (63.41%)	2152 (56.13%)	< 0.001

*Note*: The percent column displays the percentage of observations in each category out of the valid total number with non-missing values; a chi-square test was used to compare the results between the two groups

^a^continuous variable showing mean and standard deviation, and two groups of continuous data were tested using t-test

**Table 2:** Risk factors for falls in men and women: Bivariate analysis

	Men Crude OR	Women Crude OR
	(95% CI)	(95% CI)
*Environmental hazards*		
Indoor environmental hazards	1.25 (0.98–1.59)	1.53** (1.26–1.86)
Outdoor environmental hazards	1.31** (1.08–1.60)	0.99 (0.85–1.17)
*Sociodemographic*		
age (ref. 65–74 years)		
75–84 years	1.36** (1.13–1.64)	1.10 (0.94–1.28)
85+ years	1.91** (1.51–2.42)	1.45** (1.20–1.75)
Non-Hispanic White	1.38** (1.14–1.66)	1.19* (1.02–1.37)
Education (reference: Less than high school diploma)		
High school graduate	0.86 (0.69–1.08)	0.72** (0.61–0.86)
Some college	0.83 (0.64–1.08)	0.81* (0.66–1.00)
Bachelor’s degree or advanced degree	0.86 (0.68–1.07)	0.76* (0.61–0.94)
No job or retirement	1.24 (0.98–1.58)	1.51** (1.17–1.95)
Home ownership	1.08 (0.88–1.34)	0.69** (0.59–0.80)
Household income	0.89 (0.75–1.07)	0.77** (0.67–0.88)
House type (ref. single house)		
Duplex or townhouse	0.77 (0.55–1.09)	1.12 (0.88–1.43)
Mobile home	1.15 (0.80–1.66)	1.34 (0.98–1.83)
Multi-family house	0.72* (0.53–0.99)	1.11 (0.91–1.37)
*Health*		
Poor health	2.53** (1.89–3.39)	3.01** (2.33–3.89)
Fear of falling	4.07** (3.36–4.92)	2.96** (2.57–3.42)
Balance impairment	5.01** (4.16–6.03)	3.83** (3.31–4.43)
Mobility limitation within a quarter mile	3.75** (3.09–4.56)	2.38** (2.06–2.74)
Vision impairment	1.75** (1.29–2.37)	1.99** (1.60–2.48)
Arthritis	1.96** (1.66–2.32)	1.92** (1.66–2.23)
Number of medical conditions		
1	1.14 (0.79–1.64)	1.17 (0.82–1.68)
2	1.61** (1.15–2.28)	1.85** (1.33–2.58)
3	2.05** (1.45–2.90)	2.2** (1.58–3.08)
≥ 4	3.31** (2.36–4.65)	3.42** (2.46–4.75)
Depression	2.21** (1.83–2.67)	1.92** (1.66–2.21)
Medication use	2.10** (1.74–2.54)	2.17** (1.88–2.52)
*Behavior*		
Gait speed	0.45** (0.34–0.58)	0.51** (0.42–0.64)
Use of a walking aid	3.83** (3.17–4.64)	2.40** (2.08–2.78)
Frequency of going outside	0.41** (0.31–0.54)	0.64** (0.54–0.75)
Walk for exercise	0.56** (0.48–0.67)	0.59** (0.52–0.68)

*Note*: **p < 0.01, *0.01 ≤ *p* < 0.05 †0.05 ≤ *p* < 0.1

**Table 3:** Risk factors for falls by gender: multivariate analysis

	Adjusted OR
	(95% CI)
***Men***	
Outdoor environmental hazards	1.34* (1.02–1.75)
Non-Hispanic White	1.60** (1.22–2.08)
No job or retirement	0.69** (0.51–0.92)
Fear of falling	1.81** (1.38–2.37)
Balance impairment	2.80** (2.15–3.65)
Use of a walking aid	1.82** (1.31–2.52)
Walk for exercise	0.78* (0.62–0.98)
***Women***	
Indoor environmental hazards	1.37* (1.04–1.79)
Non-Hispanic White	1.45** (1.18–1.79)
Poor health	1.50* (1.03–2.18)
Fear of falling	1.59** (1.31–1.92)
Balance impairment	2.43** (1.99–2.98)
Depression	1.23* (1.02–1.49)
Medication use	1.40** (1.15–1.69)
Frequency of going outside	1.34* (1.06–1.71)
Walk for exercise	0.81* (0.68–0.97)

*Note*: **p < 0.01, *0.01 ≤ p < 0.05 †0.05 ≤ p < 0.1; after adjusted for all covariates, including age, Non-Hispanic White, education, no job or retirement, home ownership, household income, house type, poor health, fear of falling, balance impairment, mobility limitation, vision impairment, arthritis, number of medical conditions, depression, medication use, gait speed, use of a walking aid, frequency of going outside, and walk for exercise

**Table 4:** Interaction terms between environmental hazards and gender: Multivariate analysis

	Model 1 OR (95% CI)	Model 2 OR (95% CI)	Model 3 OR (95% CI)
Female	1.04 (0.91–1.20)	1.05 (0.91–1.22)	1.14 (0.98–1.33)
Indoor environmental hazards	1.30* (1.06–1.60)	1.35 (0.99–1.83)	1.29* (1.05–1.59)
Outdoor environmental hazards	0.99 (0.84–1.18)	0.98 (0.83–1.17)	1.27 (0.98–1.63)
Female × Indoor environmental hazards	–	0.94 (0.63–1.39)	–
Female × outdoor environmental hazards	–	–	0.65** (0.47–0.90)
Constant	0.13** (0.08–0.19)	0.12 (0.08–0.19)	0.12** (0.07–0.18)
N	5313	5313	5313
AIC	5682.56	5684.46	5677.91
McFadden R2 (%)	9.8	9.8	9.9

*Note*: **p < 0.01, *0.01 ≤ p < 0.05 †0.05 ≤ p < 0.1; after adjusted for all covariates, including age, Non-Hispanic White, education, no job or retirement, home ownership, household income, house type, poor health, fear of falling, balance impairment, mobility limitation, vision impairment, arthritis, number of medical conditions, depression, medication use, gait speed, use of a walking aid, frequency of going outside, and walk for exercise

## Results

### Participants’ characteristics

Characteristics of the study population are presented in Table 1. Of the 6680 eligible participants, 6672 responded to the fall-related questions. Overall, 29.6% of the participants reported any fall within a year. The prevalence of falls was significantly higher among women (31.8%) than among men (26.6%). There were gender differences in sociodemographic, health, and behavioral factors. In our study, women were more likely to be older whereas men were more likely to have a higher education level, have a job, and own a home. There was also a gender difference in household income. In terms of house type, women were more likely to live in a multi-family house whereas men were more likely to live in a single-family house. Women also reported a higher prevalence of most of the health problems, including fear of falling, balance impairment, mobility limitations, vision impairment, comorbidities, and depression (*p* < .001). In terms of walking-related behavior, women tended to walk slow and use a walking aid to go outside, but they were less likely to go outside and walk for exercise compared to men. Finally, there were no significant gender differences in relation to the indoor and outdoor environmental hazards. Most interviewers reported no environmental problems inside the home and outside the home. Only 13.7% reported any of indoor environmental hazards while 21.9% reported any of outdoor environmental hazards.

### Risk factors of falls by gender: bivariate analysis

Table 2 shows the results from the bivariate associations of each variable with the odds of falls among men. The presence of outdoor environmental hazards was significantly associated with falls in men but not in women, whereas the presence of indoor environmental hazards was significantly associated with falls in women only but not in men. Additionally, most of the health and behavioral variables were significantly associated with falls both among women and men. There were also some gender differences in sociodemographic variables associated with falls and more individual risk factors falls were founded in women. For example, education, job status, and home ownership were significantly associated with falls in women only, whereas house type was significantly associated with falls in men only.

### Risk factors of falls by gender: Multivaraite analysis

Table 3 presents the results from the multivariate models for men and women, respectively. Men-specific analysis showed that the odds of having falls within a year were 1.34 times greater for older men who lived in the areas with the presence of any outdoor environmental hazards (OR = 1.34, 95% CI = 1.02–1.75) than those who lived in areas with no environmental hazards after adjusting for all covariates. In addition to outdoor environmental hazards, the following factors were significantly associated with increased falls in men only: having no job status, using walking aid, and not engaging in walking for exercise. On the other hand, the female-specific analysis showed that the odds of having falls were 1.37 times higher for older women who lived in areas with the presence of any indoor environmental hazards (OR = 1.37, 95% CI = 1.04.-1.79) than those who did not. Additionally, having poor self-rated health status, depression, frequent medication use, and frequent going outside remained significantly associated with falls in women but not in men. However, some individual factors remained significantly associated with falls in both men and women. For example, those who were Non-Hispanic White, had a fear of falling, had balance impairment, and did not engaged in walking for exercise were more likely to have increased odds of falling than those did not in both men and women.

### Including the interaction between environmental factors and gender

**Fig. 1:**
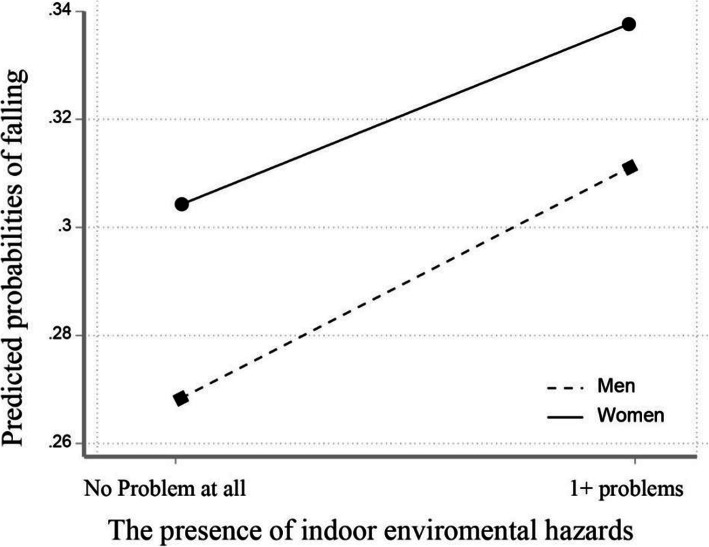
Interaction effect of indoor environmental hazards by gender on the predicted probabilities of falling

**Fig. 2:**
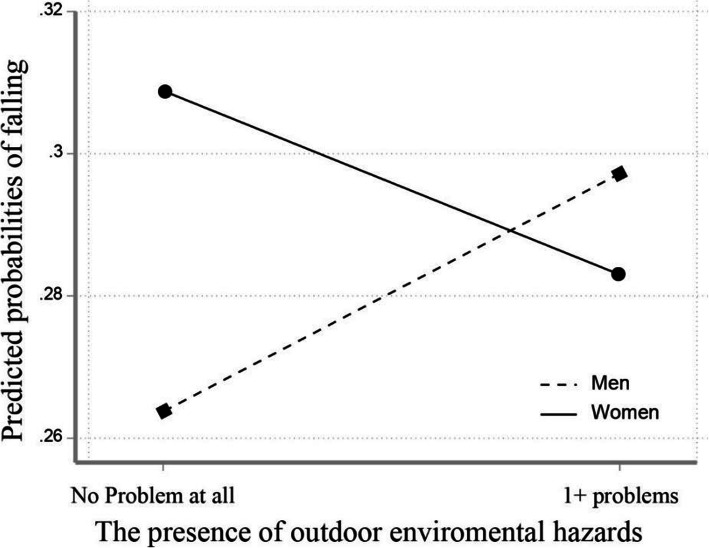
Interaction effect of outdoor environmental hazards by gender on the predicted probabilities of falling

Table 4 shows the results from the final multivariate logistics regression analysis with the full data set after considering the interaction between the built environment variable and gender to see if environmental hazards and falls differ by gender. In the base model without consideration of any interaction terms (Model 1), older people living in the presence of indoor environmental hazards (OR = 1.3, 95% CI = 1.06–1.6) reported higher odds of falling after controlling for all covariates. Model 2 and Model 3 added interaction terms for indoor environmental hazard and outdoor environmental hazard respectively by gender. The interaction term between indoor environmental hazard and gender was not statistically significant in Model 2. However, Model 3 showed a significant interaction term between outdoor environmental hazards and gender (OR = 0.65, 95% CI = 0.47–0.90), indicating that the gender difference in falling is greater when there is the presence of outdoor environmental hazards and the model fit of model 3 became slightly better than the base model. Figures 1 and 2 show the predicted probabilities of falls by gender in terms of each indoor and outdoor environmental hazard. The parallel lines on an interaction plot in Fig. 1 showed that the probability of falling increased with the presence of indoor environmental hazards in both men and women. While the probability of falling increased with the presence of outdoor environmental hazards in men, the probability of falling slightly decreased with the presence of outdoor environmental hazards in women in Fig. 2.

## Discussion

Gender differences in falls have been reported in many previous studies of older adults. However, environmental hazards associated with falls by gender are not well understood. The purpose of this study was to examine the gender differences in the associations of indoor and outdoor environmental hazards with the odds of falls. We found that indoor environmental hazards were more likely to influence the odds of falls among women while outdoor environmental hazards were more important factors influencing falls among men. Although we were unable to capture where falls occurred across gender, this study comprehensively incorporated each indoor and outdoor environmental hazards variable in the study. To the best of our knowledge, this study provides the first gender-specific prevalence of falls in relation to the indoor and outdoor environmental attributes. This study contributes to the literature on gender differences in the associations between falls and environmental hazards among older adults.

Our results confirm previous findings that older men were less likely to fall than older women [36]. Such gender disparities in fall incidence might reflect the differences in underlying sociodemographic, health, and behavioral factors. We found that individual health conditions were more associated with falls in women than in men. For example, poor self-rated health status, depression, medication use, and frequency of going outside were significantly associated with falls in women but not in men after adjusted for all variables, which was also similar findings reported by other studies [13]. Such symptoms were known to influence different lifestyles, physical activities, and living arrangement, which may result in the risk of falls in women especially while staying at home [37].

Gender-specific analysis in our study indicated that there were gender differences in the relationship between the environmental hazards and falls. On one hand, the finding showed that indoor environment was significantly associated with elevated falls for women, implying that older women may experience falls due to the complex factors between individual factors and indoor environmental hazards. This may be because compared to older men, older women tend to have more individual health problems with age and spend more time inside the home doing household activities. Previous studies have also supported that older women frequently experienced falls in the kitchen while preparing meals and washing dishes [38]. For men, indoor environmental hazards were not associated with falls, but outdoor environmental hazards were found to be an important predictor of the odds of experiencing falls even after adjusting for individual factors. This implies that older men are more likely to be influenced by outside home environments, such as problems with an uneven pavement as they walk home, for example. It may be because compared to women, men not only tend to go outside more frequently and do outdoor activities, but they may be less likely to pay attention to the areas in front of and around the home, such as the garden, access path, and neighborhood streets. This result was identified when looking at the presence of an interaction term between environmental factors and gender. We found a significant interaction between gender and outdoor environmental hazard in this study, indicating that the presence of outdoor environmental hazards increased the risks of falling in men but marginally decreased the risks of falling in women shown in Fig. 2. It is not clear if women may not go outside due to increased outdoor environmental hazards, but previous studies have reported that women were more likely to restrict their outdoor physical activity due to fear of falling outside, compared to men [39]. This result suggests that increased outdoor environmental hazards may block the possibility of falling outside among older women or rather increase their falls influenced by indoor environmental hazards, which may warrant further investigation.

Despite the importance of general home modification and maintenance of street conditions in preventing falls, few solid methods are available to evaluate fall-related environmental hazards in different locations, which makes it difficult to determine which environmental factors from the home to the neighborhood boundary could influence fall incidents. In the present study, we were able to consider the environmental hazards in different locations (i.e., indoor environment- inside the home and outdoor environment- in front of and around the home). However, we were unable to capture various environmental hazards and barriers for older fallers in the study. For example, path conditions, change in level, and obstructions around the home and even neighborhood environments were missing in our study. Although our study would contribute to the understanding of environmental factors causing falls by using limited variables, further study will need to develop comprehensive indoor and outdoor environmental checklist and incorporate life-space mobility, a concept for assessing spatial patterns of the mobility of older adults [40], to understand where a person experiences falls from the home environment to the neighborhood environment.

The strengths of this study include examining the use of multiple environmental hazards in both indoor and outdoor locations and how the hazards impact falls in older adults by gender. Most previous studies have exclusively focused on either indoor or outdoor environments, but this study enhances our understanding of environmental contributions to the risk of falling by considering indoor and outdoor environments simultaneously. In addition, this study used data from a large population-based sample of community-dwelling older adults, which helps increase the generalizability of the results. Although the number of environmental variables captured in this study were limited, the variables were relatively objective given that they were measured by an interviewer using an environmental checklist.

This study has several limitations. First, this study was based on a cross-sectional design with relatively old but baseline data which limits our ability to determine causal relations of the environment on falls across genders. Due to some missing items and selection bias due to loss to follow-up in the latest assessment dates, we decided to test indoor and outdoor environmental hazards associated with falls by gender by using baseline data although it is unclear if there is no obvious change in behavior and environmental hazards over 10 years. Further research should will consider the prospective relationship between environmental hazards and falling. Second, there were no data on where the reported falls occurred (e.g., kitchen, living room, yards, streets, or parks), which does not allow us to explain the exact mechanism of the characteristics of falls by location as well as by gender. It would be helpful to collect information about the locations of falls and the activities of the older adults when they fell in a future study. Third, we measured each built environmental hazard by indoor and outdoor, but the built environments, especially outdoor environments, are highly influenced by neighborhood socioeconomic status and quality, which was not considered in our study. We also acknowledge that this study only considered the negative environmental characteristics potentially causing risk of falling but a further study should consider the positive environmental characteristics that can help prevent falls and promote each safe indoor and outdoor activities, such as grab bars and handrails inside the home and quality of trees and maintenance of street conditions outside the home. Another limitation is that we were unable to capture how much time each participant spent in different locations although this study included a variable of frequency of going outside (days per a week) as a proxy variable for the time spent outdoors. Future studies should collect detailed information of frequency of time spent and types of activities in different locations to further understand the differences in fall incidents among men and women in responding to different built environments. Finally, further study needs to include the perceived environmental characteristics because it is reasonable to expect that the same physical environment may be perceived differently by different participants.

### Implications for practice or policy

Findings from the study provide several implications for fall prevention strategies and highlight the need for tailored approaches for men and women. First, indoor environmental hazards, including broken furniture or lamps, flooring issues, and other tripping hazards inside the home, may be an important indicator for an elevated risk of falls regardless of gender as older adults spend most of their time inside the home. Checking environmental hazards in different locations may be useful in identifying and targeting high-risk population who may require both clinical and environmental interventions to prevent falls. Second, there are few prevention programs that have incorporated gender-specific environmental strategies that consider gender differences in spatial use and behaviors. The study suggests the importance of a gender-specific environmental context of older adults and evidence regarding a differential effect of environmental relations on falls for women and men, which could potentially be used for environmental and gender-specific educational interventions to prevent falls. Finally, environmental problems could be easily modifiable and environmental interventions can be effective in reducing the risk of falls.

## Conclusion

Any older adult can experience falls at home settings and near their home. This study revealed gender differences in environmental risk factors for falls. The presence of indoor environmental hazards was an indicator of the fall risk for women but not for men while the presence of outdoor environmental hazards was an indicator of the fall risk for men but not for women. Although the present study was unable to capture where falls occur, this study can help us understand how indoor and outdoor environment could differently influence falls across gender groups. Understanding these differences and gathering information about the different risk factors across genders could help improve healthy aging promotion programs and develop effective interventions to mitigate the risks of falling among community-dwelling older adults across genders.

## Supplementary Information

**Additional file 1 Appendix Table 1.** Robust check: Construction of indoor and outdoor environmental hazards variables.

## Data Availability

The data supporting the conclusions of this article are available at https://www.nhats.org/

## Abbreviations

NHATS: National Health and Aging Trends Study
U.S.: United States
