# Supplementary material for: Falls associated with indoor and outdoor environmental hazards among community-dwelling older adults between men and women
Source: BMC Geriatr. 2021 Oct 12;21:547. doi: 10.1186/s12877-021-02499-x (PMC8507100; doi:10.1186/s12877-021-02499-x)
Supplement: Supplementary file 1 — Additional file 1 Appendix Table 1. Robust check: Construction of indoor and outdoor environmental hazards variables. [file 12877_2021_2499_MOESM1_ESM.docx]

**Appendix Table 1.** Robust check: Construction of indoor and outdoor environmental hazards variables

|  | **Men** | **Women** |
| --- | --- | --- |
|  | **Adjusted OR** | **Adjusted OR** |
|  | **(95% CI)** | **(95% CI)** |
|  |  |  |
| ***Binary variable (0: no problem, 1: 1+ problems)*** |  |  |
| Indoor environmental hazards | 1.26 (0.91-1.75) | 1.36* (1.03-1.78) |
| Outdoor environmental hazards | 1.35* (1.03-1.77) | 0.82 (0.65-1.02) |
| AIC | 2305.35 | 3398.81 |
| ***Count variable (number of problems) ^a^*** |  |  |
| Indoor environmental hazards | 1.07 (0.86-1.32) | 1.28** (1.08-1.53) |
| Outdoor environmental hazards | 1.19* (1-1.41) | 0.89 (0.78-1.02) |
| AIC | 2308.46 | 3396.96 |
| ***Index (PCA) ^b^*** |  |  |
| Indoor environmental hazards | 1 (0.92-1.1) | 1.11** (1.03-1.2) |
| Outdoor environmental hazards | 1.11* (1-121) | 9.94 (0.87-1) |
| AIC | 2308.74 | 3396.64 |
|  |  |  |

Note: a: We treated the count variable as continuous. B: A principal component analysis (PCA) was performed in Stata using varimax rotation based on an eigenvalue of 1. All variables were considered to load as the factor loading of each variable was at least .40.
